# Utilization of Blood Culture in South Asia for the Diagnosis and Treatment of Febrile Illness

**DOI:** 10.1093/cid/ciaa1322

**Published:** 2020-12-01

**Authors:** Caitlin Hemlock, Stephen P Luby, Shampa Saha, Farah Qamar, Jason R Andrews, Samir K Saha, Dipesh Tamrakar, Kashmira Date, Ashley T Longley, Denise O Garrett, Isaac I Bogoch

**Affiliations:** 1 Applied Epidemiology, Sabin Vaccine Institute, Washington, DC, USA; 2 Stanford University, Stanford, California, USA; 3 Child Health Research Foundation, Department of Microbiology, Dhaka Shishu Hospital, Sher-E-Bangla Nagar, Dhaka, Bangladesh; 4 Aga Khan University, Karachi, Pakistan; 5 Bangladesh Institute of Child Health, Dhaka Shishu Hospital, Sher-E-Bangla Nagar, Dhaka, Bangladesh; 6 Dhulikhel Hospital, Kathmandu University Hospital, Dhulikhel, Nepal; 7 Global Immunization Division, U.S. Centers for Disease Control and Prevention, Atlanta, Georgia, USA; 8 National Foundation for the Centers for Disease Control and Prevention, Atlanta, Georgia, USA; 9 Department of Medicine, Division of Infectious Diseases, University of Toronto, Toronto, Ontario, Canada

**Keywords:** blood culture, fever, typhoid, South Asia, antimicrobial resistance

## Abstract

**Background:**

Blood culture is the current standard for diagnosing bacteremic illnesses, yet it is not clear how physicians in many low- and middle-income countries utilize blood culture for diagnostic purposes and to inform treatment decisions.

**Methods:**

We screened suspected enteric fever cases from 6 hospitals in Bangladesh, Nepal, and Pakistan, and enrolled patients if blood culture was prescribed by the treating physician. We used generalized additive regression models to analyze the probability of receiving blood culture by age, and linear regression models to analyze changes by month to the proportion of febrile cases prescribed a blood culture compared with the burden of febrile illness, stratified by hospital. We used logistic regression to analyze predictors for receiving antibiotics empirically. We descriptively reviewed changes in antibiotic therapy by susceptibility patterns and coverage, stratified by country.

**Results:**

We screened 30 809 outpatients resulting in 1819 enteric fever cases; 1935 additional cases were enrolled from other hospital locations. Younger outpatients were less likely to receive a blood culture. The association between the number of febrile outpatients and the proportion prescribed blood culture varied by hospital. Antibiotics prescribed empirically were associated with severity and provisional diagnoses, but 31% (1147/3754) of enteric fever cases were not covered by initial therapy; this was highest in Pakistan (50%) as many isolates were resistant to cephalosporins, which were commonly prescribed empirically.

**Conclusions:**

Understanding hospital-level communication between laboratories and physicians may improve patient care and timeliness of appropriate antibiotics, which is important considering the rise of antimicrobial resistance.

Blood culture is the currently accepted standard for diagnosing suspected cases of bacteremia. In most high-income countries, blood cultures are routinely drawn for febrile patients when bacteremia is suspected. However, in resource-constrained settings, clinicians may choose to treat patients with antibiotics empirically, in part due to limited or absent laboratory capacity [1, 2]. In the current era of increasing antimicrobial resistance (AMR), particularly in South Asia [3, 4], blood cultures are an increasingly important tool to guide appropriate treatment and promote antimicrobial stewardship [5].

With a global action plan set forth by the World Health Organization to combat AMR [6], and an awareness of how AMR affects achieving Sustainable Development Goals [7], a better understanding of the interaction between clinician decision-making, blood culture use, and prescribed antimicrobials can help guide interventions to address AMR in South Asia. Antimicrobial susceptibility results from blood cultures can help drive clinical decisions, including choosing an appropriate antibiotic if one is required [2]. However, prior research demonstrates underutilization of blood cultures among febrile patients in low- and middle-income countries (LMICs) [8], and among patients ultimately found to be bacteremic [9]. In many LMICs, blood cultures might not be ordered for febrile cases when bacteremia is suspected for several reasons, including low sensitivity of the assay [10], high cost, and logistical issues related to patient follow-up. Indeed, the long duration to available results might dissuade clinicians from ordering cultures in settings such as outpatient departments, where many patients might not return or be contactable for follow-up.

In addition to diagnostic testing, clinicians might not revise patient therapy in response to culture results [9], though data on this topic are lacking in LMICs. In South Asia, the burden of resistant organisms is high among gram-negative organisms [11–13], including organisms that cause enteric fever (*Salmonella enteria serotypes* Typhi and Paratyphi A) [14, 15]. Of particular concern is the recent discovery and spread of extensively drug-resistant (XDR) *Salmonella* Typhi, a strain resistant to 5 different classes of antimicrobials [16, 17]. However, it is unclear if there have been meaningful changes to clinician behavior in the context of an increasing burden of antimicrobial resistance.

This study evaluated data from the Surveillance for Enteric Fever in Asia Project (SEAP) that characterized the burden of enteric fever in Bangladesh, Nepal, and Pakistan, including incidence rates, disease severity, and economic burden [18]. Here, we report on the utilization of blood culture for clinical decision-making related to the diagnosis and treatment of potential and confirmed bloodstream infections in these 3 South Asian countries; this included assessing if demographic characteristics and febrile burden at the hospital predicted prescription of blood cultures, predictors for receiving antibiotics before available blood culture results, and alterations to treatment regimen based on blood culture results, as well as whether identification of antibiotic resistant enteric fever resulted in alterations to treatment regimen.

## METHODS

### Study Design

SEAP was a prospective cohort study in hospitals in Bangladesh, Nepal, and Pakistan [18]. Prospective recruitment occurred from September 2016 to September 2019. This article presents 2 years of data from April 2017 through March 2019.

### Study Sites

Patients were recruited from inpatient, outpatient (including emergency departments), and hospital laboratories in 6 hospitals: Aga Khan University Hospital and Kharadar General Hospital (Pakistan); Dhaka Shishu Hospital and Shishu Sasthya Foundation Hospital (Bangladesh); Dhulikhel Hospital and Kathmandu Medical College Hospital (Nepal) (Table 1). More information on the sites is available in this supplement [19–21].

**Table 1.** Site Characteristics and Overall Descriptive Statistics of Recruited Patients, Surveillance for Enteric Fever in Asia Project (SEAP)—Bangladesh, Nepal and Pakistan, April 2017–March 2019

	*Bangladesh*		*Nepal*		*Pakistan*	
	Dhaka Shishu Hospital	Shishu Sasthya Foundation Hospital	Dhulikhel Hospital	Kathmandu Medical College and Teaching Hospital	Aga Khan University Hospital	Kharadar General Hospital
Site location	Dhaka`	Dhaka	Dhulikhel	Kathmandu	Karachi	
Catchment area population	1 million	1 million	84 000	448 000	1.2 million	687 000
Population served	Urban	Urban	Peri-urban/Rural	Urban	Urban	Urban
Population recruited	Pediatric (≤15)	Pediatric (≤15)	Pediatric and adult	Pediatric and adult	Pediatric and adult	Pediatric and adult
Level of adherence to recruitment protocol	Full	Partial	Full	Full	Partial	Partial
Patients recruited at outpatient	6949	12 187	1631	2020	4597	3425
Median age, (IQR)	3 (1.7–6)	3 (1.5–6)	20 (6–38)	20 (10–31)	20 (3–39)	4 (1.6–11)
Male sex, n (%)	3771 (54)	6741 (55)	902 (55)	1212 (60)	2414 (53)	1879 (55)
Prescribed blood culture, n (%)	6949 (100)	6114 (50)	1631 (100)	2020 (100)	1824 (40)	1852 (54)

### Inclusion Criteria

For prospective surveillance, patients were screened for inclusion criteria based on the point of contact with SEAP staff. At outpatient clinics and emergency departments, patients were screened for ≥3 days of fever and residence within the hospital catchment area, established by review of enteric fever patients’ addresses [22]. At inpatient wards, patients were screened for clinical suspicion or laboratory-confirmed diagnosis of enteric fever, regardless of residential address and if not enrolled at outpatient. Among those meeting SEAP inclusion criteria, study staff determined if the attending physician prescribed a blood culture. Per study protocol, only patients who had been prescribed a blood culture, and/or who agreed to provide one, were considered eligible for enrollment; patients received a blood draw shortly after enrollment (if not already provided). Patients were also enrolled retrospectively from laboratories if *Salmonella* Typhi or *Salmonella* Paratyphi A infection was confirmed by blood culture, regardless of residential address, if the patient was not already enrolled from outpatient or inpatient wards.

### Data Collection

SEAP staff collected visit date, age, and sex information on all patients meeting the inclusion criteria. If those patients were prescribed a blood culture and gave consent for enrollment, patients were interviewed about clinical information such as symptoms, disease severity, and previous care-seeking behavior. Medical records were also reviewed to obtain data on diagnoses, medication types and dates, laboratory analyses, and hospitalization. All data were entered into a custom, SQL-based system using standardized tools.

### Laboratory Analysis

All sites utilized BACTEC™ (Becton Dickinson, Baltimore, MD) to assess for bacterial pathogens in blood collected from participants. Susceptibility patterns of *Salmonella* Typhi and *Salmonella* Paratyphi A isolates were assessed via disk diffusion using Clinical & Laboratory Standards Institute guidelines [23].

### Statistical Analysis

**Figure 1.**
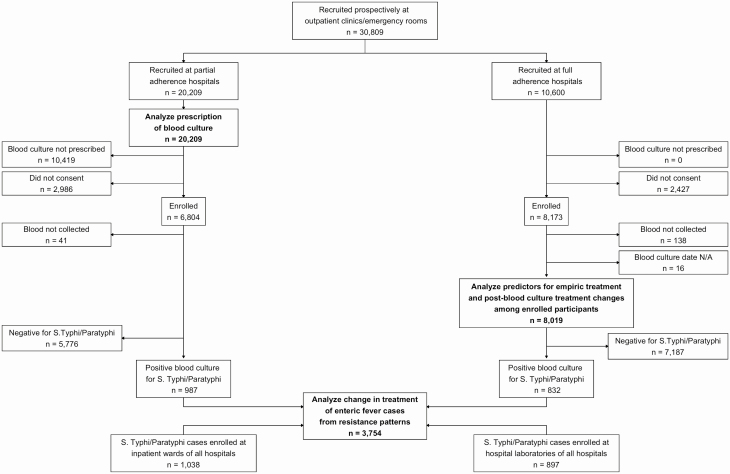
Flow of data for analysis, Surveillance for Enteric Fever in Asia Project (SEAP)—Bangladesh, Nepal and Pakistan, April 2017–March 2019. Note: Full adherence = Attending physicians prescribed a blood culture for outpatients meeting the inclusion criteria (3 or more days of fever and residence within the hospital catchment area); Partial adherence = Attending physicians only prescribed a blood culture based on clinical suspicion among those meeting the inclusion criteria.

We separated our analyses based on adherence to the recruitment protocol (Figure 1). At Dhaka Shishu Hospital (Bangladesh), all outpatients meeting the inclusion criteria were prescribed a blood culture. At both hospitals in Nepal (Dhulikhel Hospital and Kathmandu Medical College and Teaching Hospital), the local Institutional Review Board permitted SEAP staff to ask patients who had not been prescribed blood culture by physicians if they would consent to provide a blood sample. Therefore, Dhaka Shishu Hospital (Bangladesh), Dhulikhel Hospital (Nepal), and Kathmandu Medical College and Teaching Hospital (Nepal) had full adherence to SEAP recruitment protocol. At Shishu Sasthya Foundation Hospital (Bangladesh), Aga Khan University Hospital (Pakistan), and Kharadar General Hospital (Pakistan), physicians only prescribed blood culture based on clinical suspicion, therefore did not fully adhere to the SEAP recruitment protocol (subsequently referred to as “partial adherence”).

Since all recruited patients at full adherence hospitals were prescribed a blood culture, we utilized data from partial adherence hospitals for the analyses on prescription of blood culture. To evaluate if the physician’s decision-making was associated with age of the patient, we used generalized additive regression models stratified by hospital. We also assessed whether physicians’ practice for prescribing blood cultures was associated with current and/or prior febrile illness burden in the outpatient wards. We used linear regression models to estimate the association between the proportion of febrile patients prescribed a blood culture in a calendar month and the number of febrile cases recruited at outpatient wards in the same month. We then created lagged febrile case burden variables using the number of febrile cases in the previous 1–5 months and assessed in 5 additional models, stratified by hospital (18 total models).

To analyze clinical decision-making in regards to treatment before and after blood culture results, we used data from consenting participants at the 3 full adherence hospitals, since patient enrollment at partial adherence hospitals was based on clinician suspicion. We used univariate and multivariate logistic regression to evaluate predictors of patients receiving a prescription for antibiotics before blood culture results were available.

To review the effects of antimicrobial nonsusceptibility on treatment coverage, we analyzed the susceptibility patterns of confirmed enteric fever cases compared with antibiotics prescribed before and after blood culture results were available at both full and partial adherence hospitals (Figure 1). We performed this analysis using *χ*^2^ tests grouped by country, as hospitals within each country were either in the same catchment area or within 25 km of each other.

All statistical tests were performed in R version 3.6.1 and were considered significant at *P* < .05.

### Ethical Considerations

Study protocols were reviewed by Institutional Review Boards in the United States (Centers for Disease Control and Prevention), Bangladesh (Bangladesh Institute of Child Health Ethical Review Committee), Nepal (Nepal Health Research Council Ethical Review Board Approval), and Pakistan (National Bioethics Committee), in addition to local hospital ethical review boards.

## RESULTS

During the study period, 30 809 patients from all hospitals were recruited at outpatient clinics. The majority were male, and the median age differed by country, as well as by catchment area within country (Table 1). Among the 20 209 recruited at hospitals with partial adherence, 9790 (48%) were prescribed a blood culture by the treating physician; this differed by hospital (*P* < .001), with the lowest proportion over the study period at Aga Khan University Hospital (40%).

**Figure 2.**
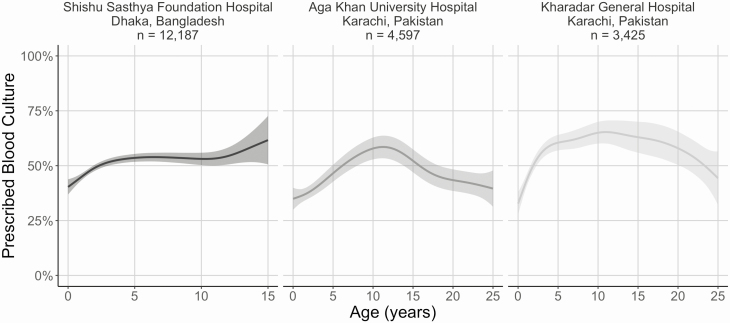
Probability of receiving a prescription for blood culture, by age in years and site, (SEAP)—Partial adherence* hospitals in Bangladesh and Pakistan, April 2017–March 2019. *Hospitals where physicians prescribed a blood culture based only on clinical suspicion and did not fully adhere to the SEAP recruitment protocol of prescribing a blood culture to all patients with fever for 3 more days and residence within the hospital catchment area.

At all three partial adherence hospitals, the probability of being prescribed a blood culture varied with age (Figure 2). At Shishu Sasthya Foundation Hospital, the probability was highest at 5 years old, and remained relatively constant until 15 years, but at Aga Khan University Hospital and Kharadar General Hospital, the probability was highest between 10 and 15 years. There was no difference in receiving a blood culture at Aga Khan University Hospital and Kharadar General Hospital comparing males with females, but at Shishu Sasthya Foundation Hospital, males had 10% higher odds (95% CI, 3%–19%).

**Figure 3.**
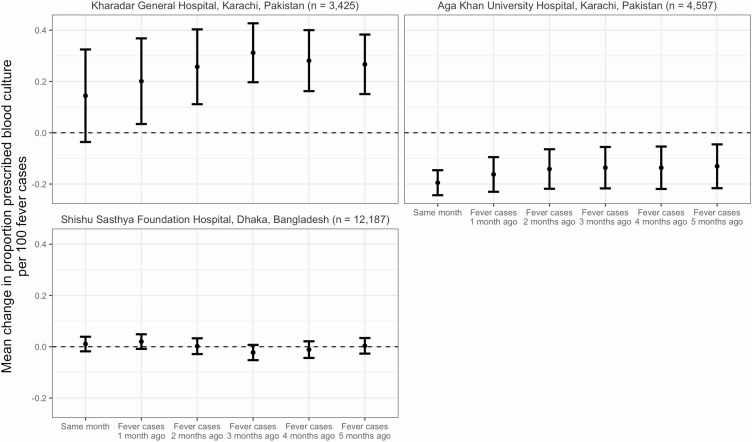
Proportion of febrile outpatients prescribed a blood culture predicted by total number of febrile outpatients in the same month and 1–5 months ago, (SEAP)—Partial adherence* hospitals in Bangladesh and Pakistan, April 2017–March 2019. *Hospitals where physicians only prescribed a blood culture based on clinical suspicion and did not fully adhere to the SEAP recruitment protocol of advising a blood culture to all patients with fever for 3 or more days and residence within the hospital catchment area.

Physicians’ practice for prescribing blood cultures based on the burden of febrile illness at outpatient wards also varied by hospital (Figure 3). At Kharadar General Hospital, the proportion of febrile outpatients prescribed a blood culture was not significantly predicted by the number of febrile cases in the same month (mean change per 100 cases, .14 [95% CI, −.04–.32]). However, when compared with the number of febrile cases in the previous 1–5 months, case counts from 3 months prior had the strongest association, with a 31% mean increase (95% CI, .20–.43) in the proportion prescribed a blood culture per increase in 100 febrile cases. At Aga Khan University Hospital, the association was negative; an increase of 100 febrile cases predicted a 19% decrease (95% CI, −.24–−.15) in the proportion prescribed a blood culture, and the association was weaker compared with case counts from the previous 1–5 months. At Shishu Sasthya Foundation Hospital, increases in the number of cases in the same month or the previous 1–5 months did not predict an increase or decrease in the proportion prescribed a blood culture. We also did not observe seasonal patterns in physicians prescribing blood culture for febrile illness.

Among the 10 600 outpatients recruited at full adherence hospitals, 8019 (76%) were enrolled, had blood collected, and had available blood culture date data. Among those, 6355 (79%) were prescribed antibiotics empirically. Patient sex did not predict receiving antibiotics empirically, but older participants had slightly higher odds when adjusted for site (OR [age in years] 1.01, 95% CI, 1.005–1.014). Patients with female heads of the household with postsecondary education had significantly lower odds of receiving antibiotics empirically compared with no education, while patients who reported taking antibiotics previously and were febrile at presentation had significantly higher odds. Patients provisionally diagnosed with fever of unknown origin or viral fever had significantly lower odds of receiving antibiotics empirically, while patients provisionally diagnosed with enteric fever, lower respiratory tract infection, and urinary tract infection had significantly higher odds. Notably, patients provisionally diagnosed with upper respiratory tract infection had the highest independent odds of receiving empiric antibiotics empirically among diagnoses examined (OR 5.26, 95% CI, 2.97–10.2) (Table 2).

**Table 2.** Independent Predictors of Febrile Patients Receiving Antibiotics Prior to Available Blood Culture Results, Surveillance for Enteric Fever in Asia Project (SEAP) —Full Adherence^e^ Hospitals in Bangladesh and Nepal, April 2017–March 2019

	No EmpiricAntibiotics Prescribedn = 1664 (%)	EmpiricAntibiotics Prescribedn = 6355 (%)	Unadjusted OR (95% CI)	Adjusted OR (95% CI)
**Demographics**				
Age in years, median (IQR)	8 (3–21)	5 (2–13)	.989 (.986–.993)	**1.01 (1.005–1.014)** ^a^
Male sex	950 (57)	3534 (56)	.94 (.84–1.05)	1.00 (.89–1.12)^a^
Education level of female head of household	n = 804	n = 3271		
None	166 (21)	710 (22)	Ref.	Ref.
Primary	275 (34)	1279 (39)	1.09 (.88–1.34)	.92 (.73–1.16)^a^
Secondary	182 (23)	678 (21)	.87 (.69–1.1)	.94 (.73–1.21)^a^
Postsecondary	181 (23)	604 (19)	.78 (.62–.99)	**.69 (.54–.89)** ^a^
**Severity**				
Days to seek care, median (IQR)	n = 1659, 3 (2–5)	n = 6344, 4 (3–5)	.99 (.97–1)	.99 (.96–1.01)^b^
Previous antibiotics taken	438/1640 (27)	2240/6221 (36)	1.54 (1.37–1.74)	**1.55 (1.29–1.87)** ^b^
Days unable to perform usual activities, median (IQR)	n = 1659, 3 (.5–4)	n = 6338, 2 (0–4)	.97 (.95–.98)	1.00 (.98–1.03)^b^
Febrile at presentation (≥99.5°F)	391/1662 (24)	2223 (35)	1.75 (1.55–1.98)	**1.52 (1.26–1.83)** ^b^
Admitted	55/1662 (3)	448/6352 (7)	2.22 (1.68–2.98)	**2.69 (1.73–4.35)** ^c^
**Provisional diagnosis** ^d^, (row%)				
Enteric fever	439 (12)	3363 (88)	3.13 (2.78–3.53)	**2.01 (1.59–2.54)** ^c^
LRTI	28 (5)	564 (95)	5.68 (3.95–8.53)	**1.63 (1.23–2.18)** ^c^
URTI	177 (14)	1065 (86)	1.69 (1.43–2.01)	**5.26 (2.97–10.2)** ^c^
Fever of unknown origin	180 (40)	269 (60)	.36 (.3–.44)	**.59 (.42–.83)** ^c^
UTI	40 (12)	299 (88)	2 (1.45–2.84)	**2.51 (1.41–4.8)** ^c^
Viral fever	286 (48)	307 (52)	.24 (.21–.29)	**.22 (.17–.29)** ^c^
**Site**, (row%)				
Dhaka Shishu Hospital	680 (14)	4074 (86)	Ref.	Ref.
Dhulikhel Hospital	244 (17)	1204 (83)	.82 (.7–.97)	**.65 (.47–.89)** ^c^
Kathmandu Medical College and Teaching Hospital	740 (41)	1077 (59)	.24 (.21–.27)	**.24 (.18–.31)** ^c^

^a^Adjusted for site.

^b^Adjusted for site, demographics, and severity markers (except admission status).

^c^Adjusted for site, demographics, severity markers, and other diagnoses.

^d^Reference categories for diagnoses are absence of diagnosis of interest.

^e^Hospitals where physicians prescribed a blood culture to all patients with fever for three of more days and residence within the hospital catchment area, based on the SEAP recruitment protocol.

**Figure 4.**
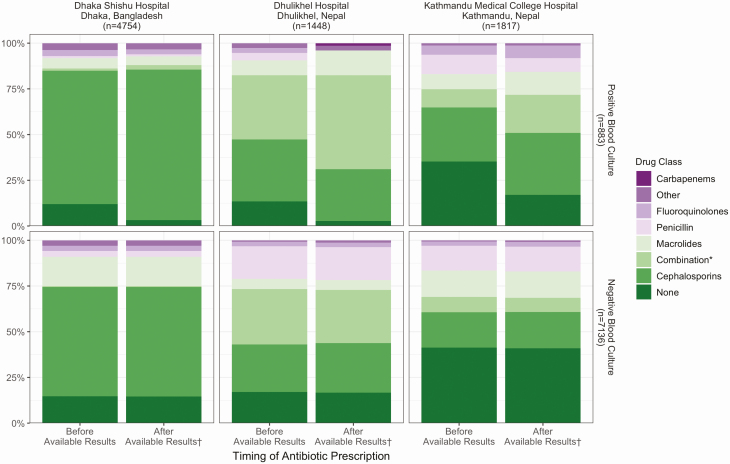
Antibiotics classes prescribed before and after results available from blood culture, by blood culture status and hospital (SEAP)—Full adherence^‡^ hospitals in Bangladesh and Nepal, April 2017–March 2019. ‡ Hospitals where physicians prescribed a blood culture to all patients with fever for 3 or more days and residence within the hospital catchment area, based on the SEAP recruitment protocol. †If no change in treatment, empiric prescription displayed. *Multiple antibiotics prescribed at given time point.

The choice of antibiotic class given before and after available blood culture results differed among the 3 full adherence hospitals (Figure 4). At Kathmandu Medical College and Teaching Hospital, 59% of febrile outpatients received antibiotics empirically, compared with 86% at Dhaka Shishu Hospital and 83% at Dhulikhel Hospital. Among those who received antibiotics empirically, 72% received cephalosporins at Dhaka Shishu Hospital, while at Dhulikhel Hospital most patients received a combination of 2 or more drugs (37%) or cephalosporin monotherapy (32%). At Kathmandu Medical College and Teaching Hospital, 34% empirically treated patients received cephalosporins, followed by macrolides (23%) and penicillin (22%). After blood culture results were available, 196/883 (22%) patients positive for any pathogen and 100/7136 (1.4%) of patients with negative blood cultures received additional antibiotics. At Dhaka Shishu Hospital, most blood culture positive patients with no antibiotics prescribed empirically were switched to cephalosporin therapy. This resulted in 82% of positive patients and 63% of all patients prescribed cephalosporins after available blood culture results. At Dhulikhel Hospital, combinations of 2 or more drugs were the most common (51%) treatment regimen for patients with positive blood cultures, followed by cephalosporin monotherapy (28%). At Kathmandu Medical College and Teaching Hospital, a variety of antibiotics were prescribed for patients with positive blood cultures, with cephalosporins representing the most prevalent class (34%), followed by a combination (21%).

**Figure 5.**
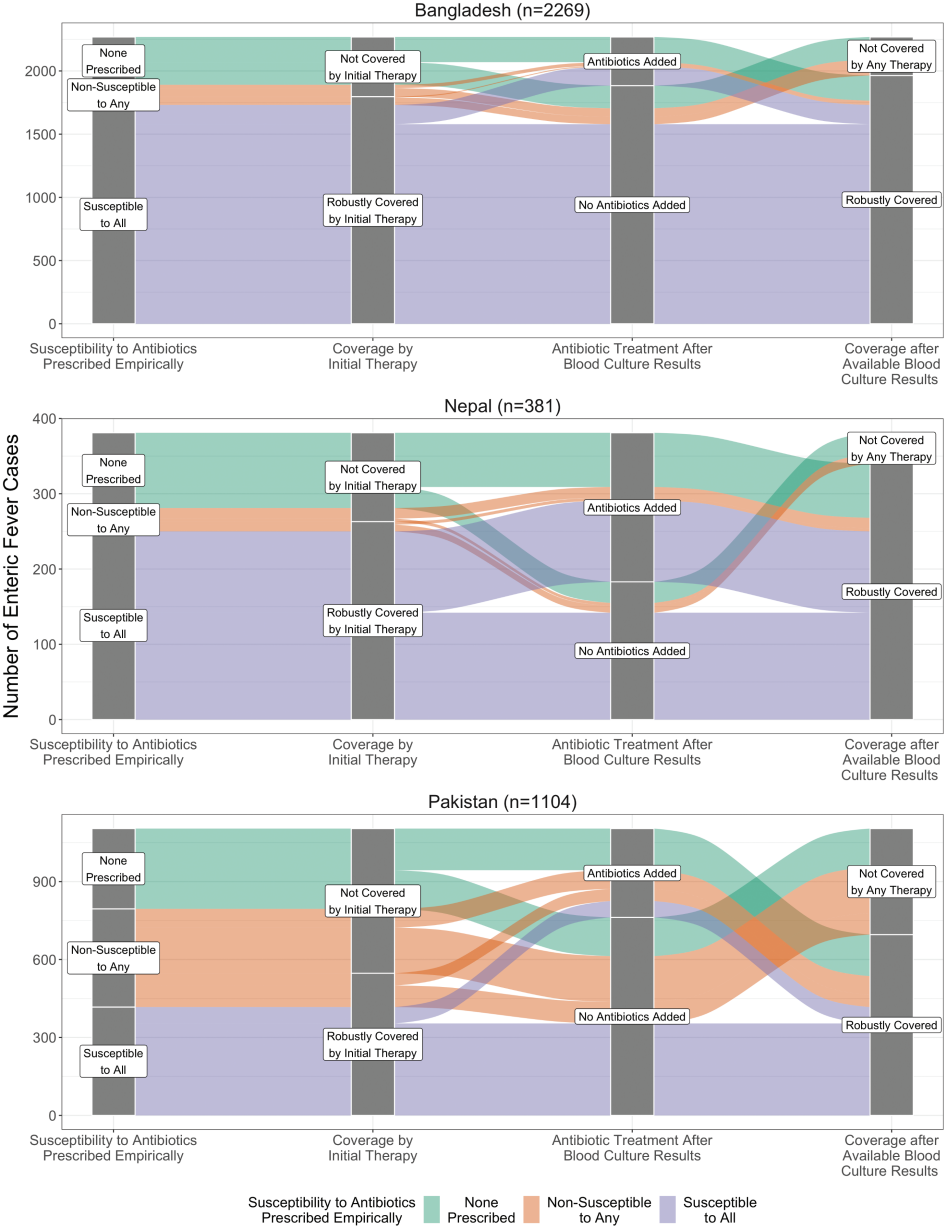
Changes to treatment after results of blood culture and antimicrobial sensitivity testing among participants with *Salmonella* Typhi and Paratyphi A isolated, by country, (SEAP)—Bangladesh, Nepal and Pakistan, April 2017–March 2019. Note: This alluvial diagram depicts all enteric fever cases stratified by country and how treatment was modified based on blood culture results. The colors originate at the first stage, where patients were categorized based on their isolate’s susceptibility pattern to antibiotics prescribed empirically, if any. Based on the antibiotic, whether multiple antibiotics were prescribed empirically, and isolate susceptibility, the second stage stratifies patient as covered or not covered by initial therapy. The third stage depicts whether patients were prescribed additional antibiotics based on their initial therapy coverage, resulting in the fourth stage—whether a patient’s final treatment status with the hospital resulted in coverage.

The six hospitals and all recruitment locations had a total of 3754 enteric fever cases. Of all enteric fever cases, 2967 (79%) received antibiotics empirically, and among those, 569 (19%) were prescribed an antibiotic that the strain they were infected with tested nonsusceptible (Figure 5). This varied by country (Bangladesh: 8%, Nepal: 11%, Pakistan: 48%, *P* < .001). However, 209 (37%) of those were still covered because multiple antibiotics were empirically prescribed. Among the 1147 (31%) not covered by initial therapy because antibiotics had not been empirically prescribed (n = 787), or with discordant therapy due to nonsusceptibility (n = 360), 544 (47%) had antibiotics added, which also varied significantly by country (Bangladesh: 48%, Nepal: 73%, Pakistan: 42%, *P* < .001). This ultimately resulted in a total of 20% of all enteric fever cases without antibiotic coverage: 9% because no antibiotics were prescribed, and 11% because the discordant therapy was not changed (Bangladesh: 8%/6%, Nepal: 7%/3%, Pakistan: 13%/23%, *P* < .001).

## Discussion

Our data revealed that clinical decision-making, both in the utilization of blood cultures for diagnosis and antimicrobial treatment of potential blood stream infections, may not be reflective of epidemiologic and microbiologic data made available by cultures. This is shown in the varying patterns of changes in physicians’ clinical decision-making in ordering blood cultures based on hospital-level burden data. Blood cultures were also differentially utilized based on age, with the probability of receiving a blood culture not reflective of recent age-stratified burden estimates for enteric fever in Bangladesh and Pakistan, generated by SEAP (unpublished). Additionally, while results from blood cultures may drive changes in patient therapy, broad spectrum antibiotics were utilized most often, even after microbial confirmation from blood culture. Yet, antibiotics prescribed empirically did not always provide robust coverage of bacteremic patients, especially in outbreak situations with transmission of resistant pathogen strains.

While blood cultures remain the primary method and current standard for diagnosing bloodstream infections, this test is utilized less in South Asian settings for suspected blood stream infections than recommended in clinical guidelines in high-income countries [24]; less than half of cases with 3 days of fever received a blood culture at hospitals with partial adherence, which may still be higher than the usual proportion without study protocols in place.

We also found 3 different scenarios at the partial adherence hospitals of how physicians responded to the burden of febrile disease. At Aga Khan University Hospital in Pakistan, we found that as the number of febrile cases increased, the proportion of these cases prescribed blood culture decreased and this association was strongest in the same month; this would suggest that any burden data may not be affecting diagnostic decision-making and any increase in febrile cases may be suspected to be nonbacteremic illness. At Shishu Sasthya Foundation Hospital in Bangladesh, we found no association between increases in febrile cases and the proportion prescribed blood culture. While viral fever outbreaks are common [25, 26], enteric fever accounted for 63% of blood cultures in one study in Dhaka [27], so enteric fever may be commonly suspected regardless of any changes to the burden of febrile cases. At Kharadar General Hospital in Pakistan, the epicenter of an XDR typhoid outbreak in Karachi, we found that febrile burden data from 3 months prior most strongly predicted changes in the proportion of febrile cases prescribed blood culture.

Our study also demonstrated a wide range of clinical practice in the choice to prescribe empiric antibiotics and the antibiotic regimens chosen prior to the availability of blood culture data. Such diversity of practice was seen even within the same country. Empiric treatment choices were typically driven by clinical suspicion, however recent data demonstrates that clinical diagnoses of febrile syndromes have low positive predictive value [28]. We noted that patients that reported previously taking antibiotics were more likely to receive antibiotics empirically while patients from more educated households were less likely, potentially reflecting healthcare-seeking behavior by education of household; another analysis of SEAP data notes that patients from poorer households initially sought care for febrile illness from pharmacies [22]. In this study, most febrile patients were empirically treated with third-generation cephalosporins, and, after blood culture and sensitivity data became available, a further proportion were prescribed a third-generation cephalosporin or a combination of antibiotics. Initiatives to tailor antibiotics using results from antimicrobial susceptibility patterns, rather than maintaining or broadening treatment in these scenarios, might help improve patient outcomes [29, 30], but are difficult considering the scope of resistance among enteric fever, which has left few oral antibiotics available for treatment. Disadvantages in using broad-spectrum antibiotics compared to narrow-spectrum have been shown, such as selection for resistant bacteria and harmful effects to the microbiome, which are especially disruptive during childhood [31].

For patient-level clinical decisions, we demonstrated that while blood culture results can help improve the proportion of culture-confirmed enteric fever patients with treatment coverage, many without an effective empiric regimen are still not prescribed an antimicrobial agent that matches culture results, despite the available blood culture and susceptibility testing results. There are several possible reasons why this gap in care exists: clinicians not following up with laboratory data; outpatients not available or not returning to hospital for their laboratory results; physicians not changing treatment choices due to clinical improvement; or system-wide processes that hinder communication between the laboratory, physicians, and patients. This gap in care can lead to delays in initiating appropriate antimicrobial therapy for bloodstream infections, which have been shown to be associated with greater morbidity and mortality [32–34]. This association has been further demonstrated with SEAP data, where patients with a longer duration between symptom onset to healthcare presentation, and patients with XDR typhoid, were more likely to be hospitalized [35]. While the case fatality rate in the SEAP study was low [35], the study population resided within endemic areas and may have more immunity resulting from previous exposures; improving gaps in care such as the ones elicited in this study could be important for outbreak situations in nonendemic areas.

There were several limitations to our analysis. As the data were a secondary analysis within the SEAP study, we were only able to review febrile cases that resided within the study-specified hospital catchment area, which may represent a less severe population than all febrile cases presenting to the hospital, or those who had 3 or more days of fever, which may represent a more severe population. It is unclear how this inclusion criteria may have affected our estimates, but should be considered in the interpretation of the study population. We were also only able to review associations between number of febrile cases and proportion prescribed blood culture aggregated by month, which is likely not representative of individual-level decision-making and the variety of factors that go into individual patient care. This is especially true considering we did not have wealth, severity, or clinical suspicion data on those not prescribed a blood culture. Another limitation of our analysis is that treatment was ascertained via chart review and some prescriptions could be missing from the charts and not included in our analysis, lowering the proportion of enteric fever cases reported as covered with an appropriate antibiotic. However, SEAP staff routinely performed quality control and assurance on all clinical information, including types and dates of prescription, and this would not affect the proportional breakdown of medications prescribed.

## Conclusions

These data demonstrate that blood culture diagnostics have the potential to improve the clinical management of patients with febrile illness in South Asian settings, but reaching this goal may require more than just performing a blood culture test. A productive area for potential improvements in patient care could be efforts to understand factors driving clinical decision-making, especially during outbreaks of pathogens with antimicrobial resistance, as appropriate response to hospital-level burden information may impact patient-care decisions. Even though the hospitals participating in the study had strong laboratory capacity, the processes to inform treating physicians were less operationalized in all of these settings. Optimizing communication strategies, as well as developing a systems approach to facilitate better coordination, could also contribute to this. As seen with our data, blood culture and antimicrobial susceptibility testing can help to ensure those with bacteremia are treated with an appropriate antimicrobial regimen in a timely manner. Future efforts to establish a low-cost, rapid test with high sensitivity and specificity are needed to contribute to the appropriate narrowing of the antimicrobial spectrum. Considering the increasing antimicrobial resistance in these settings, blood culture should be utilized more effectively in the present to guide clinical practice, especially where laboratory capacity is already established.

## Notes

***Acknowledgments.*** The authors would like to thank all involved with the Surveillance for Enteric Fever in Asia Project, and especially to the participants and the research staff at participating hospitals and laboratories in Nepal, Bangladesh, and Pakistan.

***Financial support.*** This work was supported by a grant from the Bill and Melinda Gates Foundation (grant number INV-008335).

***Supplement sponsorship.*** This supplement is sponsored by the Sabin Vaccine Institute and made possible by a grant from the Bill & Melinda Gates Foundation.

***Potential conflicts of interest.*** Authors report grants from the Bill and Melinda Gates Foundation during the conduct of the study. Kashmira Date also reports receiving funds through a conditional gift agreement between the Sabin Vaccine Institute (primary grantee for the Surveillance for Enteric Fever in Asia Project grant from the Bill and Melinda Gates Foundation) and the CDC Foundation. Samir Saha reports grants from the World Health Organization, Bill & Melinda Gates Foundation, GlaxoSmithKline, Pfizer, Sanofi Pasteur, and Edinburgh University, outside the submitted work. All other authors have no potential conflicts to disclose. All authors have submitted the ICMJE Form for Disclosure of Potential Conflicts of Interest. Conflicts that the editors consider relevant to the content of the manuscript have been disclosed.
